# Global Research Trends in the Latarjet Procedure: A Bibliometric and Visualized Study

**DOI:** 10.3390/medicina58081008

**Published:** 2022-07-28

**Authors:** Fabriccio J. Visconti-Lopez, Akram Hernández-Vásquez, Diego Azañedo, Jose Fernando Sanchez Carbonel

**Affiliations:** 1 Department of Health Sciences, Universidad Peruana de Ciencias Aplicadas, Lima 15023, Peru; fabricciovisco@gmail.com (F.J.V.-L.); jose.sanchez@tum.de (J.F.S.C.); 2 Centro de Excelencia en Investigaciones Económicas y Sociales en Salud, Vicerrectorado de Investigación, Universidad San Ignacio de Loyola, Lima 15024, Peru; 3 Facultad de Ciencias de la Salud, Universidad Científica del Sur, Lima 15067, Peru; dazanedo@cientifica.edu.pe; 4 Department of Sports Orthopaedics, Technical University of Munich, 80333 Munich, Germany

**Keywords:** bibliometrics, orthopaedic procedures, trends, orthopedics

## Abstract

*Background and Objectives*: Latarjet is among the procedures indicated to treat shoulder instability, producing excellent results, including low instability rates and high patient satisfaction. The aim of this study was to report the characteristics of scientific articles that address the subject of the Latarjet procedure through the use of bibliometric analysis. *Materials and Methods*: Bibliographic searches were performed for original articles published in journals indexed by the Web of Science database until 2021, with no language restrictions. *Results*: A total of 668 articles published in 87 journals were included. The first publication was in 1981; the most registered publications were in 2018 and 2021 (89 articles), with an annual percentage growth rate of 11.9. Provencher MT was the author with the most published articles, and the institutional affiliation with the most original articles was the Steadman Philippon Research Institute. The most cited article was a study by Burkhart and Beer, and the scientific journal with the most publications on the subject was the Journal of Shoulder and Elbow Surgery. Most published studies included keywords such as dislocation, instability, and meta-analysis. *Conclusion*: There has been a sustained increase in original articles on the Latarjet procedure. However, the greatest growth in articles has occurred during the last decade, demonstrating the considerable interest among the world scientific community.

## 1. Introduction

Shoulder instability is a pathology that encompasses both subluxation and dislocation of the glenohumeral joint. It is estimated that approximately 1–2% of the general population will experience a glenohumeral dislocation in their lifetime [1]. The pathophysiological mechanism of this injury was described in 1923 by Bankart, who also described the Bankart technique to repair the anterior inferior labral detachment observed in such cases [2]. In chronic anterior shoulder instability, between 41% and 86% of cases involve glenoid bone loss, and 6% involve glenoid bone loss of 25% or more [3]. In this regard, the literature mentions that glenoid injuries with bone loss of more than 25% must be reconstructed with a bone transfer or augmentation and not only by repairing the labral injury [3,4].

Latarjet is among the procedures indicated for performing glenoid bone grafts. This technique was first described in 1954. Since then, a series of adaptations of the original technique has been described, as well as arthroscopic methods [5]. The Latarjet procedure considerably improves anterior glenohumeral stability by augmenting the anterior inferior glenoid bone defect and reconstructing the anterior capsule [6]. This technique has been reported to produce excellent results, with low instability rates (2.0–4.9%) and 98% patient satisfaction [7,8]. The advances in this technique make it a minimally invasive procedure performed by arthroscopy, conferring improved benefits, such as the ability to address other pathologies, improved aesthetics, decreased soft tissue alteration, a lower infection rate, shorter recovery times and lower complication rates [6].

Knowledge and understanding of the advances in the literature on a particular technique, such as Latarjet, are essential to guide funding and research priorities and identify potential opportunities for collaboration or training [9]. Bibliometric analysis is a useful tool for mapping publications and evaluating performance in specific research fields [10,11]. Unlike narrative reviews, this type of analysis provides quantitative and statistical analyses to assess the development of scientific disciplines, thereby precluding susceptibility to researcher bias [12]. For example, such studies include citation analyses, which make it possible to assess the impact of a journal, article, author or new research topic, observing its evolution through a historical perspective in clinical practice [13,14].

It is estimated that scientific articles in the field of orthopaedics, as well as on the Latarjet procedure, have grown exponentially over the years. Identifying the most relevant research topics the authors who publish most on the topic could be helpful for informational or collaborative purposes [15]. Therefore, the objective of this study was to report the characteristics of scientific articles that address the subject of the Latarjet procedure through the use of bibliometric analysis. Additionally, an analysis of the organizations, countries and individuals responsible for the most scientific production, as well as an analysis of keywords, was carried out.

## 2. Materials and Methods

### 2.1. Source of Bibliometric Data and Search Strategies

A bibliometric analysis of original articles published in journals indexed in the Web of Science (WOS) was performed. As one of the largest bibliometric databases, the WOS is a bibliographic database with temporal coverage since 1975 and a total of 90 million records, and it is the database most frequently used in bibliometric studies [10].

The search query used to search in title, abstract, author keywords, and keywords plus fields was TS = (Latarjet*). We considered all articles, regardless of the language in which they were written. The search term was based on the search strategies of previous systematic reviews on the Latarjet procedure [16,17]. All bibliometric data were extracted from the WOS database in one day (6 December 2021). Other document types, such as reviews, meeting abstracts, letters and corrections, were excluded.

### 2.2. Data Export and Study Selection

The obtained data were exported to RIS format and imported into the Rayyan web application [18], in which one of the authors (FJVL) independently reviewed and selected the titles and abstracts of each of the records that met the inclusion criteria. The inclusion criteria were: (1) original articles addressing the Latarjet procedure and (2) published until December 2021. From each of the records not included, the WOS «Accession Number» was extracted to exclude them from the initial search and obtain the final complete records for bibliometric analysis.

### 2.3. Text Cleaning

Prior to bibliometric analysis, the data were standardized manually in the fields of author, institutional affiliation and keywords to eliminate redundancies and inconsistencies by creating thesauri in txt format under the two-column format (label and replace by), as recommended in the VOSviewer software (Leiden, Netherlands) manual version 1.6.17 [19]. For example, separate registries with authors, such as “agneskirchner, jens d.” and “Agneskirchner, J.D.”, were combined and unified as “agneskirchner, j.d.”. Similarly, all variants of the keywords “latarjet intervention”, “latarjet surgery”, “latarjet-procedure” and “latarjet technique” were combined and unified as “latarjet procedure”.

### 2.4. Statistical Analysis

The authors described this statistical analysis previously [20,21]. Microsoft Excel 2019 (Washington, IL, United States) was used for descriptive analysis, graph plotting and to investigate the trend of publication growth over the years. We analysed the total publications; total journals; total citations; number of articles published annually; 10 articles with the most citations; journals with the most publications; and coauthorship networks according to the authors, institutional affiliations and keyword co-occurrence network [20,21]. The bibliometric indices were obtained using the Bibliometrix package in the R programming language [22]. In addition, the free, open-source science mapping software VOSviewer 1.6.17 (Leiden, Netherlands) [19] was used to visualize the coauthorship network, co-organisation network, and co-occurrence network analysis of keywords. The link in the coauthorship/organisation network represented authors’ or organisations’ collaborations, and larger nodes indicated more publications by an author/organisation. For keyword analysis, we extracted the keywords plus, and the co-occurrence network represented that the relationship between items is built according to the quantity of publications in which they occur together. We used the fractional counting method for network analyses for documents with a minimum of 2 documents and a maximum of 25 authors per author, as well as the association normalization method, repulsion of the nodes of 2, and attraction of the nodes of 1. Finally, for the keyword co-occurrence network, a threshold of two mentions for co-occurrence in titles and abstracts was used.

### 2.5. Ethical Considerations

All the data utilised in this work were downloaded from bibliographic databases; therefore, neither ethics committee approval nor informed consent was required.

## 3. Results

**Figure 1:**
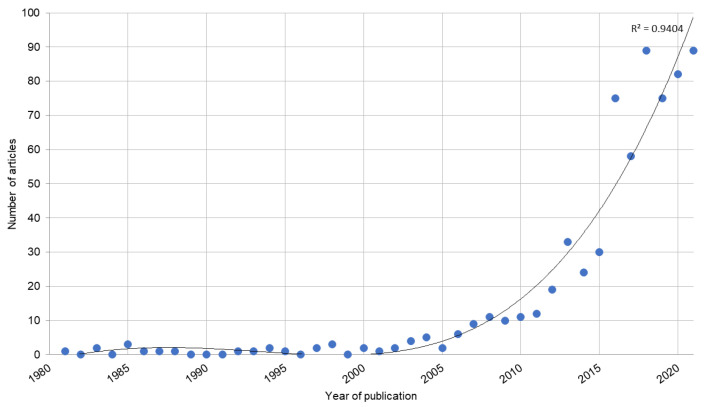
Trend in the publication of articles on the Latarjet procedure in Web of Science from 1981 to 2021.

A total of 668 publications on the Latarjet procedure were identified in the WOS. According to the WOS citation report, until 2021, 250 articles (times cited: 2819, average number of citations per item: 11.28) were open-access, and 418 articles (times cited: 11,008, average number of citations per item: 26.15) were non-open-access. The first publication on the subject was published in 1981, and the maximum number of publications occurred in the years 2018 and 2021, with a total of 89 publications in each year and an annual percentage growth rate of 11.9 (Figure 1).

**Figure 2:**
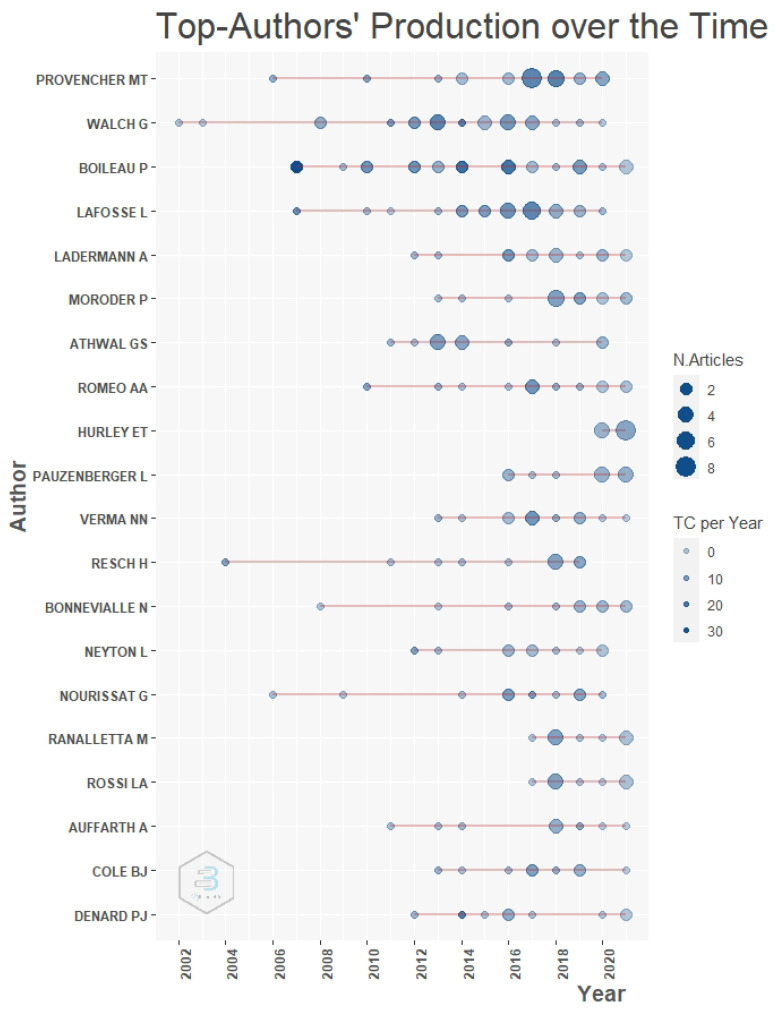
Visualization of the production of the top authors over time. Abbreviation: TC, total citations.

The authors with the most publications were Provencher M.T. (the Steadman Clinic, Vail, United States) and Walch G. (Centre Orthopédique Santy/France), with 27 and 25 publications, respectively. Provencher MT began publication in 2006, unlike Walch, who began in 2002. Hurley E.T. (New York University, Langone Health, United States) is also among the top authors with the most publications, after having begun publication in 2020 and with 12 publications on the topic to date (Figure 2).

A study by Burkhart et al. published in 2000 was the most cited, with a total of 1008 citations and an average of 45.8 citations per year. The studies by Allain et al. (1998) and Balg et al. (2007) occupied the second and third position in terms of the number of citations, with a total of 432 and 425 citations, respectively, although the average number of citations per year for the study by Balg et al. was higher than that of Allain et al. (28.3 vs. 18.3). A study by Mizuno et al. was the most recently published study (2014), which, with 173 citations, is among the top 10 most cited articles (Table 1).

The Journal of Shoulder and Elbow Surgery, as well as Arthroscopy: The Journal of Arthroscopic and Related Surgery had the most articles published on the Latarjet procedure, with 94 and 67 articles, respectively. Both journals are of American origin. Additionally, among the top 10 journals with the most publications, six are from the United States, with two from Germany, one from France and one from the United Kingdom (Table 2).

Most articles were from correspondent authors from the United States (183 publications) and France (100 publications). However, the most single-country publications (SCP) articles were from the United States, with 153/183 (83.6%), whereas France had fewer SCPs, with 70/100 (70%) (Table 3). Likewise, the most total cited articles on the Latarjet procedure were from the United States (3958) and France (3297); however, the highest average number of citations per article was achieved by Sweden (94.8), followed by France (33.0) (Table 4).

**Figure 3:**
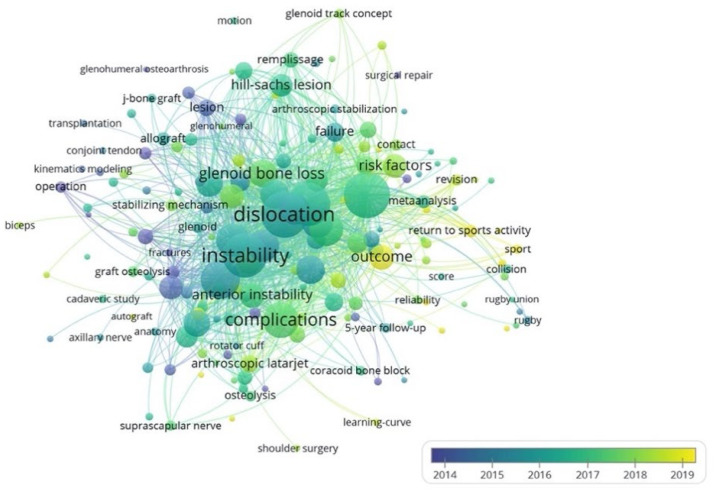
Visualization map of the keywords network. Different colours represent the average publications per year. Large circles indicate authors with the most publications.

**Figure 4:**
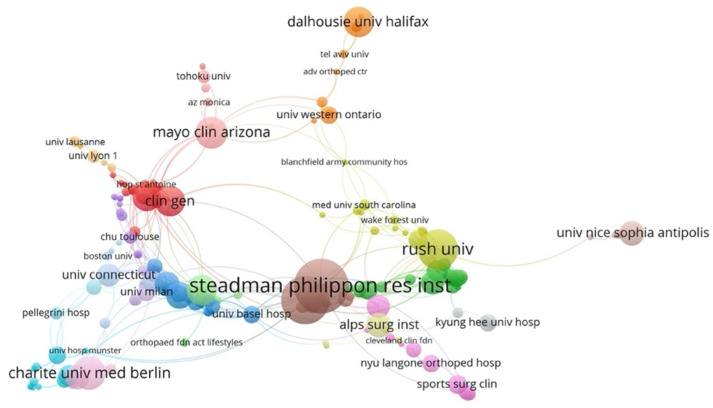
Visualization map of network of affiliations. Large circles indicate institutions with the most publications.

In relation to the keywords used, in general, the most-used terms were: “dislocation”, “instability” and “metaanalysis”, excluding the terms “latarjet procedure” and “shoulder” from the analysis. In 2014, the most-used keywords were: “lesion”, “operation” and “glenohumeral”, whereas the most common keywords in 2017 were: “dislocation” and “instability” (Figure 3). In 2019, the terms “outcomes”, “sport” and “return to sports activity” were the most common keywords. Finally, the institutional affiliations with the most publications on the Latarjet procedure were the Steadman Philippon Research Institute, the Rush University Medical Center and Charite University Medicine, Berlin (Figure 4).

## 4. Discussion

In the present study, we determined the scientific production on the Latarjet procedure in the WOS worldwide until the year 2021. There has been an increase in the number of original articles worldwide, with the most registered publications in 2018 and 2021 (89 articles). Likewise, we evaluated bibliometric indicators, such as the number of publications; trends according to year, author and institution; journals with the most articles; articles with the most citations; the number of articles according to the country of the correspondent; and countries with the most joint articles on the subject. Finally, we conducted an analysis of keywords.

In relation to the production of original articles of the Latarjet procedure, there was a notable increase within the last decade, especially in 2016, at which time production was 2.5-fold greater than in the previous year. However, we must clarify that this does not also mean an increase in the number of citations as reported in previous bibliometric analyses of related topics [31,32]. The increase in the production over the years is possibly due to the 2014 research on bone augmentation to reduce the risk of recurrent dislocation when there is a glenoid defect greater than 25% [4]. Likewise, the most original articles were published in 2018 and 2020, reaching a total of 89 articles in each year out of a total of 668 articles between 1981 and 2021. This increase in studies may be due to the fact that it is currently recommended to apply the procedure even with a lower percentage of bone loss (from 25% to 17.3%), as further loss would result in recurrent glenohumeral instability after arthroscopic Bankart repair, causing the Bankart procedure to be less considered, and the Latarjet procedure is performed more safely to avoid reintervention due to re-instability [33]. This increase in scientific production indicates considerable interest in this technique, possibly because the Latarjet procedure has shown better results than other surgical techniques. In this regard, it has been reported that the use of this technique is associated with fewer recurrences, fewer post-surgical dislocations, increased patient satisfaction and reduced motion range in rotation compared to the Bankart procedure [34].

With respect to authors, it was found that Provencher MT had the most publications related to this surgical technique, with 27 articles and an increased presence during the year 2017. This is in accordance with the institutional affiliation with the most publications, which was the Steadman Philippon Research Institute, to which this author belongs as a physician, researcher and faculty member. This institution performs difficult arthroscopic and related surgical cases, as evidenced by the publications, as well as news and press articles by Provencher [35]. In addition, this author has a long history in research, as assistant editor-in-chief emeritus of the Arthroscopy Journal: The Journal of Arthroscopic and Related Surgery, which is one of the journals with most articles published on the Latarjet procedure, with more than 200 publications and authorship of five orthopaedic books [35,36]. 

In terms of articles with the most citations, we observed that the most-cited article was by Burkhart and De Beer (2000), with a total of 1008 citations (23), followed by an article by Allain et al. (1998), with 432 citations) [24]. The article by Burkhart and De Beer, which reported the results of a series of patients with glenohumeral instability, concluded that in patients with significant glenoid bone loss, the surgeon should consider reconstruction with the Latarjet procedure, using transfer of the coracoid. On the other hand, the article by Allain et al. reported the long-term results of the Latarjet procedure, in which approximately 9 out of 10 patients were described as having an excellent or good result according to the Rowe system [37], which is a score that determines shoulder instability based on the evaluation of stability, mobility and function of this joint [38]. On the other hand, it is worth mentioning that we found a higher average number of citations per article for publications that are not open access. Although this may be contrary to the assumption that open-access publications could have more citations per publication, this was previously reported in a study evaluating the WOS database, in which the authors concluded that there is only slight variability in the number of citations according to access status [39].

As for the main scientific journals in which the original articles on the Latarjet procedure were published, we found that the Journal of Shoulder and Elbow Surgery (94 articles), followed by Arthroscopy: The Journal of Arthroscopic and Related Surgery (67 articles) and the American Journal of Sports Medicine (64 articles) had the most publications. These journals were catalogued as the most frequent publishers of studies related to shoulder arthroscopy [40,41].

This bibliometric analysis showed that the country that published the most on the subject was the United States, with 183 articles. In addition, according to our quantitative analysis, Provencher MT, the author with the most publications on this subject, is affiliated with American institutions. Likewise, from 2000 to 2015, the United States was the country that made the most economic investment in biomedical research, becoming the country with the most annual articles in scientific journals [42]. Behind the United States, the countries that published the most on the Latarjet procedure were France (70 articles) and Germany (25 articles), which is in agreement with previously reported analyses on the theme of orthopaedics and traumatology. However, no countries from Latin America and the Caribbean were found within the ranking of countries with the most publications, which could be explained by the poor investment in research made within this region compared to the other countries included within this ranking [43].

The main keywords associated with the Latarjet procedure were “dislocation” and “instability”. This can be explained by the fact that these terms are associated with the reasons why this procedure is performed [5]. Likewise, the keywords of the most recent articles included “outcome”, “sport” and “return to sports activity”, which can be justified by to the need to report and differentiate the indications between an arthroscopic treatment, such as Bankart repair, and bone transfer, such as the Latarjet procedure [44]. In addition, it is important to mention sport as a keyword to differentiate the Instability Severity Index score if the patient being treated performs competitive or recreational sports or if the sport is of contact or another category [25].

Within the limitations of the study, it should be mentioned that a bibliometric analysis is dependent on the availability of the data of the articles obtained with the search strategy. It should be noted that this study was carried out in a single database; thus, articles on the Latarjet procedure in other bibliographic databases (Medline, SciELO and Scopus) were not included in the analysis. However, the WOS is a bibliographic database with a temporal coverage since 1900 and a total of 90 million records and is therefore widely used in bibliometric studies [45].

## 5. Conclusions

Based on this quantitative bibliometric analysis, we can conclude that the Latarjet procedure is a subject of considerable interest in the scientific community, especially in the last decade, due to the exponential increase in original articles published during this period. We synthesized and described the evidence regarding information such as the top-cited authors, as well as the authors and institutions that have contributed the most publications on the subject. Thus, this study provides past and present guidance on this topic as a basis for future collaboration and research, as well as for academic purposes. Furthermore, this may represent relevant information for professionals who are seeking high-level training on this topic. Identification of the most cited articles could also help clinicians and scientists in these areas to gain an overview of past and current trends in the field of the Latarjet procedure, providing a basis for both future discussion and research.

## Figures and Tables

**Table 1:** List of top 10 most cited articles on the Latarjet procedure.

N	Article	DOI	Total Citations	Average Number of Citations per Year	NTC
1	Burkhart SS, 2000, Arthroscopy [23]	10.1053/jars.2000.17715	1008	45.8	2.0
2	Allain J, 1998, J Bone Joint Surg Am [24]	10.2106/00004623-199806000-00008	432	18.0	2.8
3	Balg F, 2007, J Bone Joint Surg Br [25]	10.1302/0301-620X.89B11.18962	425	28.3	3.2
4	Burkhart SS, 2007, Arthroscopy [7]	10.1016/j.arthro.2007.08.009	334	22.3	2.5
5	Lafosse L, 2007, Arthroscopy [26]	10.1016/j.arthro.2007.06.008	242	16.1	1.8
6	Hovelius L, 2004, J Shoulder Elb Surg [8]	10.1016/j.jse.2004.02.013	193	10.7	2.5
7	Shah AA, 2012, J Bone Joint Surg Am [27]	10.2106/JBJS.J.01830	187	18.7	3.9
8	Tauber M, 2004, J Shoulder Elb Surg [28]	10.1016/j.jse.2004.01.008	186	10.3	2.4
9	Chuang TY, 2008, Arthroscopy [29]	10.1016/j.arthro.2007.10.008	181	12.9	4.6
10	Mizuno N, 2014, J Shoulder Elb Surg [30]	10.1016/j.jse.2014.02.015	173	21.6	4.6

DOI: digital object identifier; NTC, number of total citations.

**Table 2:** List of top 10 journals with the most publications on the Latarjet procedure.

N	Journal	Frequency	Country	IF *
1	Journal Of Shoulder and Elbow Surgery	94	United States	3.507
2	Arthroscopy: The Journal of Arthroscopic and Related Surgery	67	United States	5.973
3	American Journal of Sports Medicine	64	United States	7.01
4	Arthroscopy Techniques	54	United States	NA
5	Knee Surgery Sports Traumatology Arthroscopy	49	Germany	4.114
6	Orthopaedic Journal of Sports Medicine	35	United States	3.401
7	Orthopaedics & Traumatology-Surgery & Research	25	France	2.425
8	Operative Techniques in Sports Medicine	16	United Kingdom	0.318
9	International Orthopaedics	15	Germany	3.479
10	Journal Of Bone and Joint Surgery-American Volume	15	United States	6.558

* Source: Journal Citation Reports ™ 2021. NA: not available; IF: impact factor.

**Table 3:** Top 10 countries publishing on the Latarjet procedure.

N	Country	Frequency	%	SCP	MCP
1	United States	183	27.7	153	30
2	France	100	15.1	70	30
3	Germany	41	6.2	25	16
4	Italy	32	4.8	17	15
5	Switzerland	32	4.8	11	21
6	Canada	31	4.7	22	9
7	China	29	4.4	27	2
8	United Kingdom	20	3.0	16	4
9	Austria	15	2.3	6	9
10	Japan	15	2.3	12	3

Abbreviation: SCP, single-country publications; MCP, multiple-country publications.

**Table 4:** Top 10 countries with the most total citations on the Latarjet procedure.

N	Country	Citations	Average Article Citations
1	United States	3958	21.6
2	France	3297	33.0
3	Sweden	1043	94.8
4	Canada	556	17.9
5	Italy	515	16.1
6	Switzerland	473	14.8
7	Germany	438	10.7
8	Austria	396	26.4
9	Australia	311	22.2
10	Japan	255	17.0

## Data Availability

Not applicable.
